# Supplementary figures and images for: Preliminary evaluation of computer-assisted home training for French cochlear implant recipients
Source: PLoS One. 2023 Apr 28;18(4):e0285154. doi: 10.1371/journal.pone.0285154 (PMC10146517; doi:10.1371/journal.pone.0285154)

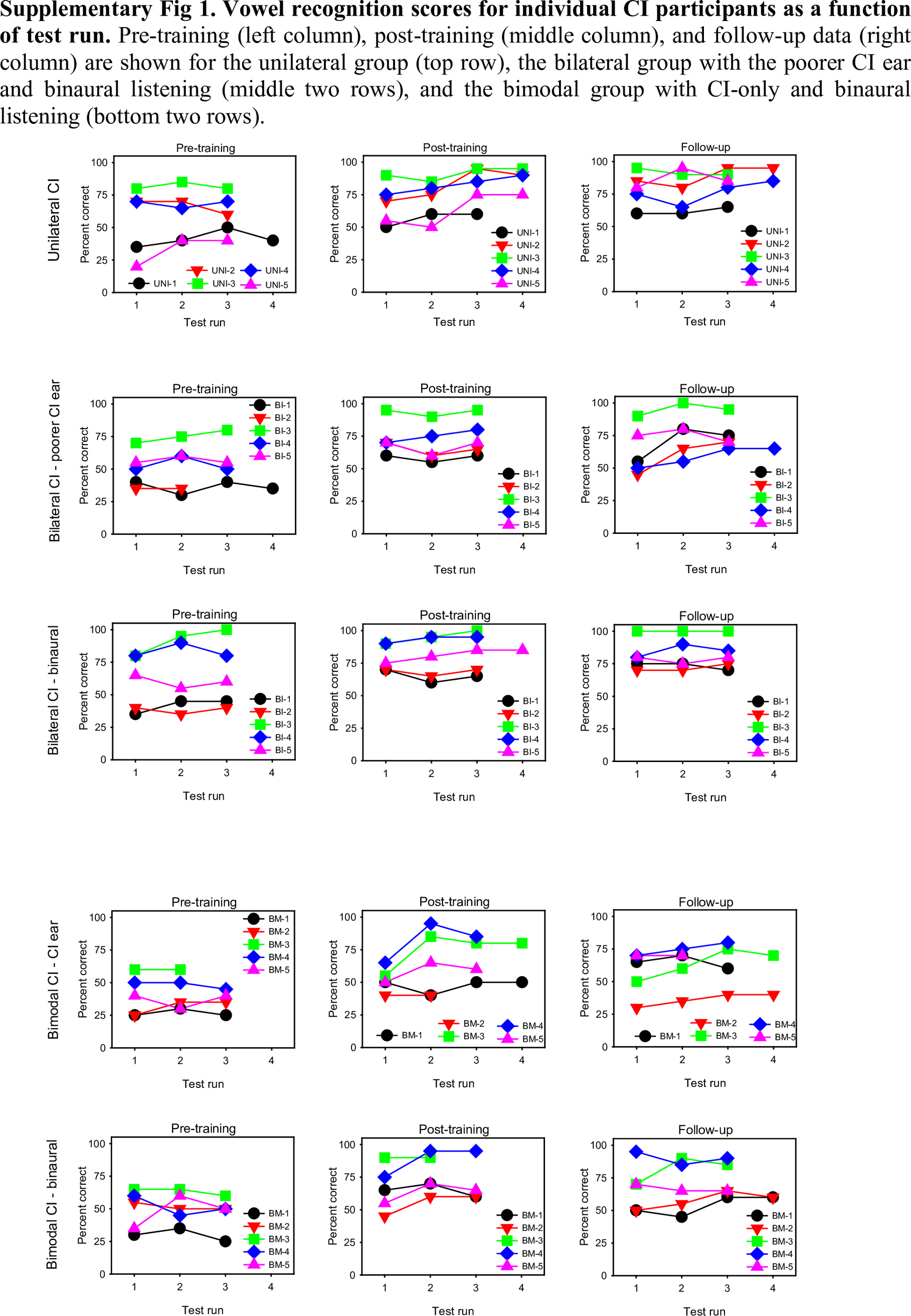

Supplement: S1 Fig — Pre-training (left column), post-training (middle column), and follow-up data (right column) are shown for the unilateral group (top row), the bilateral group with the poorer CI ear and binaural listening (middle two rows), and the bimodal group with CI-only and binaural listening (bottom two rows). (TIF) [file pone.0285154.s001.tif]

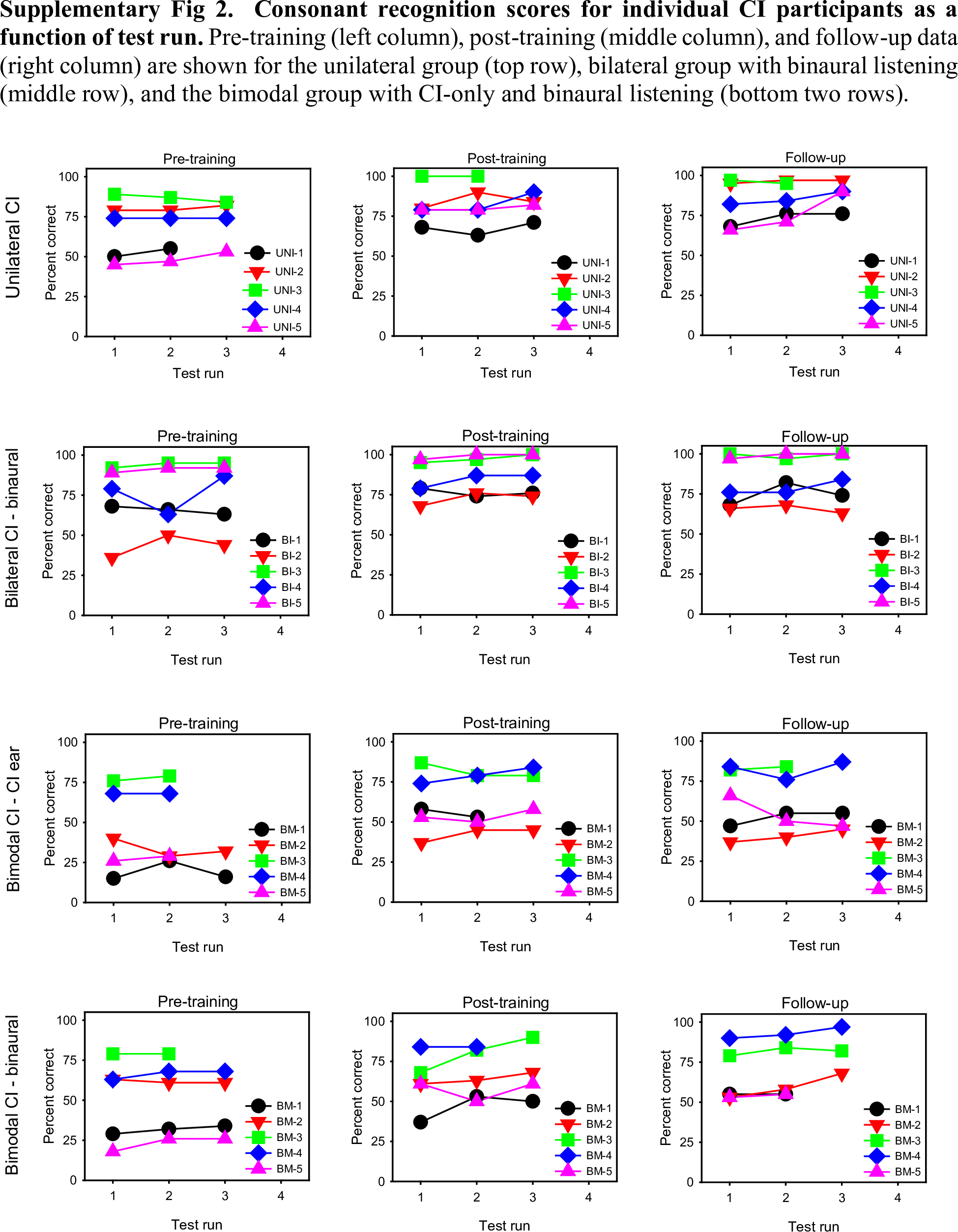

Supplement: S2 Fig — Pre-training (left column), post-training (middle column), and follow-up data (right column) are shown for the unilateral group (top row), bilateral group with binaural listening (middle row), and the bimodal group with CI-only and binaural listening (bottom two rows). (TIF) [file pone.0285154.s002.tif]
