# Supplementary material for: Preliminary evaluation of computer-assisted home training for French cochlear implant recipients
Source: PLoS One. 2023 Apr 28;18(4):e0285154. doi: 10.1371/journal.pone.0285154 (PMC10146517; doi:10.1371/journal.pone.0285154)
Supplement: Attachment — Submitted filename: Marked up manuscript.docx [file pone.0285154.s004.docx]

**Benefits of computer-assisted home training**

**for French cochlear implant patients**

Sandrine Kerneis^1^, John J. Galvin III^1,2^, Stephanie Borel^1,3^,

Jean Baqué^1^, Qian-Jie Fu^4^, David Bakhos^1,2,5^

^1^University Hospital Center of Tours, FRA

^2^House Institute Foundation, Los Angeles, CA, USA

^3^Assistance Publique Hôpitaux de Paris, Pitié-Salpêtrière and Sorbonne University, FRA

^4^Department of Head and Neck Surgery, David Geffen School of Medicine,

UCLA, Los Angeles, CA, USA

^5^INSERM UMR 1253 I-Brain, Université François-Rabelais de Tours,

CHRU de Tours, FRA.

Running title: Computer-based training for French cochlear implant patients

Corresponding author:

Sandrine Kerneis

University Hospital Center of Tours, France

2 Boulevard Tonnellé

CHRU de Tours

Hôpital Bretonneau

Service d'ORL et Chirurgie Cervico-faciale

37000 Tours

France

Tel: +33631726136

Email: sandrine.kerneis@gmail.com

**Abstract**

For French cochlear implant (CI) patients, in-person clinical auditory rehabilitation is typically provided during the first few years post-implantation. However, this is often inconvenient, it requires substantial time resources and can be problematic when appointments are unavailable. In response, we developed a computer-based home training software (“French AngelSound”) for French CI patients.

We recently conducted a pilot study to evaluate French AngelSound in 15 CI patients (5 unilateral, 5 bilateral, 5 bimodal). Outcome measures included phoneme recognition in quiet and sentence recognition in noise. Unilateral patients were tested with the CI alone. Bilateral patients were tested with each CI alone to determine the poorer ear to be trained, as well as with both ears (binaural performance). Bimodal patients were tested with the CI ear alone, and with the contralateral hearing aid (binaural performance). Patients trained at home over a one-month period (10 hours total). Phonemic contrast training was used; the level of difficulty ranged from phoneme discrimination in quiet to phoneme identification in multi-talker babble. Unilateral and bimodal patients trained with the CI alone; bilateral patients trained with the poorer ear. Outcomes were measured before training, immediately after training was completed (post-training), and one month after training was stopped (follow-up).

For all participants, post-training CI-only performance significantly improved after phoneme training with the CI ear alone. For bilateral and bimodal CI patients, binaural performance significantly improved after training with a single CI ear. Follow-up measures showed that training benefits were largely retained.

These preliminary data suggest that the phonemic contrast training in French AngelSound may significantly benefit French CI patients. As such, French AngelSound may complement clinical auditory rehabilitation for French CI patients and may be especially valuable when in-person rehabilitation is not possible.

**Keywords:** cochlear implant, auditory training

**Introduction**

After cochlear implantation, post-lingual cochlear implant (CI) patients must adapt to the new electrical speech patterns relative to central templates developed during previous acoustic hearing. Usually, the greatest adaptation occurs during the first 3-6 months of implant use, beyond which there is little further improvement [1,2,3,4]. Despite this initial adaptation, there is considerable variability in CI patient outcomes. Some patients receive little benefit from the latest technology, and many CI users have difficulty with challenging listening environments.

While passive learning (i.e., no explicit training) may drive adaptation during the first 3-6 months of CI use, computer-based auditory training (i.e., explicit feedback) has been shown to improve speech understanding, even in patients with years of CI experience [5,6,7,8,9,10,11,12,13,14,15]. Active training may better engage CI patients’ auditory plasticity and facilitate and/or accelerate adaptation. However, the method and stimuli used for auditory training also matter. Training with sentences (in quiet or in noise) largely targets top-down processes, where CI patients may rely strongly on contextual cues for speech understanding. Compared to phoneme recognition, sentence recognition has been shown to be more robust to the spectro-temporal distortion associated with CIs [16]. Training at the phoneme level may target more bottom-up processes, where feedback can help CI patients adapt to the spectro-temporal distortion (especially the acoustic-to-electric frequency mismatch) with electric hearing.

Fu and colleagues developed a computer-based research platform to provide training to English-speaking CI patients in the lab or in patients’ homes, using their own computers [5,6,7,8]. More than 4500 monosyllable words spoken by multiple talkers were recorded for the training software. Moderate amounts of monosyllable word training (~10 hours) that targeted phonemic contrasts significantly improved CI patients’ vowel and consonant recognition by ~15 percentage points, with significant improvements also observed for sentence recognition in noise [5,6,7,8,9,10,14]. Early research versions of the software developed by Fu and colleagues were modified to create “AngelSound” training software, which has been freely available for many years to CI patients, clinicians and researchers. Worldwide, the software has been requested and/or downloaded by more than 100,000 CI patients, clinicians and researchers. AngelSound is currently available in English and Mandarin language versions.

In France, post-implantation auditory rehabilitation programs are available during the first few years of CI use. These may not be ideal in that they require in-person visits with local speech therapists; during the recent COVID-19 pandemic, such non-essential in-person interactions were greatly curtailed. Methods are also not consistent across CI centers. In general, there is no theoretically based approach, and no opportunity to train within challenging listening environments such as noise or competing speech. Computer-based auditory training (as in AngelSound) allows CI patients to train at home at their convenience, with opportunities to adjust the level of difficulty according to individual patient needs (e.g., training in quiet or in noise). As noted above, moderate amounts of computer-based home training have been shown to significantly improve CI users’ vowel, consonant and sentence recognition, with an average improvement of ~15 percentage points [8]. Historically, such post-training improvements are often as large if not larger than those brought about by improvements in CI technology. While previous studies have demonstrated the efficacy of computer-based home training [5,6,7,8,9,10,11,12,14,15], no such training is currently available for French CI patients. To address this issue, we recently developed a French version of AngelSound that was similar in structure to previous English and Mandarin language versions. Stimuli consist of high-quality recordings (44.1 kHz sampling rate, 16-bit resolution) of French phonemes, words and sentences produced by multiple native French talkers.

While computer-based home training has been shown to benefit unilateral CI patients, auditory rehabilitation strategies may be different for bilateral (CI in both ears) and bimodal patients (CI in one ear, hearing aid in the other ear). For bilateral CI patients (especially for sequential implantation), speech understanding may be better in one ear than in the other. This performance asymmetry has been shown to limit the binaural benefit in bilateral CI patients when speech and noise are presented directly in front of the listener [17,18]. It is possible that the peripheral representations may differ due to different electrode positions and or patterns of neural survival in each ear, and the ear with the better representation may dominate the binaural perception [19]. When speech performance was similar across ears, the binaural benefit (i.e., gains in binaural performance relative to the better ear) was significantly greater [17,18]. For sequentially implanted CI patients, speech patterns developed with the first CI may dominate the binaural perception. Given that performance asymmetry appears to limit the binaural benefit, training the poorer ear may improve performance, reduce asymmetry, and increase binaural benefit in bilateral CI patients.

Increasing numbers of CI patients have residual acoustic hearing in the non-implanted ear. Depending on the extent of acoustic hearing in the non-implanted ear and the position and frequency allocation in the CI ear, there may be an inter-aural frequency mismatch. While unilateral CI patients may adapt to an intra-aural frequency mismatch over time, bimodal CI patients may have difficulty adapting to the mismatch in the CI ear, given the dominance of the acoustic hearing ear. When hearing is limited in the acoustic ear, there is often large performance asymmetry due to loss of high-frequency speech information. Performance asymmetry has been shown to significantly limit binaural benefit in bimodal CI patients [20]. Reducing the inter-aural frequency mismatch (which likely leads to performance asymmetry) has been shown to significantly improve binaural benefit in simulations of bimodal listening [21]. Training the CI ear alone may improve adaptation to the frequency mismatch, thereby improving integration of acoustic and electric hearing and thus, binaural benefit.

In the present study, we evaluated the efficacy of computer-based home training with French AngelSound in adult, native French-speaking unilateral, bilateral and bimodal CI patients. Outcome measures included vowel recognition in quiet, consonant recognition in quiet, and sentence recognition in noise, as well as the shortened Speech, Sound and Quality questionnaire (SSQ-12; [22]). Unilateral patients were tested with the CI alone. Bilateral patients were tested with the CI alone to determine the poorer ear to be trained and were also tested with both ears (binaural performance). Bimodal patients were tested with the CI ear alone, and with the contralateral hearing aid (binaural performance). Patients were trained at home over a one-month period (10 hours total training time). Unilateral and bimodal patients trained with the CI alone; bilateral patients trained with the poorer CI ear. Outcomes were measured before training was begun, immediately after training was completed, and one month after training was stopped. Consistent with previous studies, we expected that training with the CI alone would improve performance with the CI alone. We also expected that training with the poorer ear (bilateral CI patients) or with the CI alone (bimodal CI patients) would also improve binaural test outcomes.

**Methods**

**Ethics statement**

All participants provided written informed consent prior to enrolling in the study. The study was approved by the Ethics committee at the University of Tours (Approval number: 2019-067).

**Participants**

Fifteen adult participants (7 male, 8 female; mean age at testing = 51.1±15.3 years) were recruited from the CI patient population at the ENT center at the University Hospital Center of Tours, France between June 2020 and February 2021. Inclusion criteria were: 1) Native French speaking, 2) More than 6 months of experience with their CI, 3) Ability to use a computer, follow instructions, and train at home. Table 1 shows demographic information. Five participants were unilateral CI patients, 5 were bilateral CI patients, and 5 were bimodal CI patients (hearing aid in one ear, CI in the other ear). All participants’ CI settings were checked by an audiologist before enrolling in the study, and patients were instructed not to change these settings during the study. The audiologist explained the study to the patients, who were then referred to one of two researchers who verified inclusion criteria and obtained written informed consent and planned the three upcoming study appointments.

Sixteen NH adults (mean age at testing = 42.5±17.9 years) were also recruited to evaluate the phoneme and sentence recognition tests. A one-way ANOVA showed no significant difference in age at testing between CI and NH participants [F(1,29) = 2.0; p = 0.164].

Table 1. Demographic information for the 15 CI participants.

| **Participant** | **Age test**  **(yrs)** | **Sex** | **Hearing** | **Etiology** | **Dur deaf (yrs)** | **CI exp (yrs)** | **CI device** | **PTA** | **SAT** | **WRS** |
| --- | --- | --- | --- | --- | --- | --- | --- | --- | --- | --- |
| UNI-1 | 69 | M | Post | SHL - L Presbycusis - R | 10 | 5 | Coch | 28 | 38 | 100 |
| UNI-2 | 46 | F | Post | SHL - L Neurinoma - R | 1 | 5 | Coch | 18 | 25 | 100 |
| UNI-3 | 51 | F | Post | Unknown | 15 | 2 | Coch | 15 | 30 | 100 |
| UNI-4 | 55 | M | Post | Unknown | 29 | 6 | Coch | 27 | 30 | 100 |
| UNI-5 | 30 | F | Post | Unknown | 19 | 6 | Med-El | 35 | 47 | 60 |
| BI-1 | 55 | M | Post | Menières | L - 18  R - 28 | L - 2  R - 12 | Coch | 32 | 30 | 100 |
| BI-2 | 59 | M | Pre | Unknown | L - 55  R - 57 | L - 4  R - 2 | Coch | 27 | 32 | 100 |
| BI-3 | 18 | F | Pre | Congenital | L- 14  R -16 | L - 4  R - 2 | AB | 33 | 43 | 90 |
| BI-4 | 63 | F | Post | Ototoxicity | L - 20  R - 23 | L -10  R -7 | Coch | 18 | 28 | 100 |
| BI-5 | 26 | M | Post | Cogan syndrome | L - 0.5  R - 0.5 | L - 1  R - 1 | Coch | 27 | 27 | 100 |
| BM-1 | 69 | F | Post | Presbycusis - L  SHL - R | 5 | 3 | Coch | 27 | 27 | 100 |
| BM-2 | 60 | M | Post | Chronic  otitis | 41 | 2 | AB | 27 | 52 | 90 |
| BM-3 | 56 | F | Post | Hereditary | 21 | 5 | Coch | 33 | 33 | 100 |
| BM-4 | 49 | F | Post | Unknown | 18 | 2 | AB | 30 | 36 | 100 |
| BM-5 | 60 | M | Post | Hereditary | 6 | 2 | Coch | 13 | 24 | 100 |

CI = cochlear implant; UNI = unilateral CI; BI = bilateral CI; BM = bimodal CI; yrs = years; SNHL = sensorineural hearing loss; dur deaf = duration of deafness before CI; CI exp = CI experience; Coch = Cochlear device; AB = Advanced Bionics device; PTA = aided (CI) pure-tone average threshold (across 500 – 1000 - 2000 kHz) in dB HL (hearing level); SAT = speech audibility threshold in dB HL; WRS = percent correct monosyllable word recognition score in quiet

**Outcome measures**

Outcome measures included phoneme recognition in quiet and sentence recognition in steady noise, as well as the SSQ-12 questionnaire [22]. Baseline measures (vowel and consonant recognition in quiet, SRTs in noise) were measured before training was begun (“pre-training”). Participants then began the home training for 1 month (10 hours total training), immediately after which outcomes were measured again (“post-training”). After completing post-training measures, participants were asked to discontinue any training with French AngelSound. One month after training was stopped, outcomes were re-measured to observe whether any training benefits had been retained (“follow-up”). Fig 1 illustrates the timeline of the study.

**Fig 1. Timeline of the study.**

Due to the generally high variability in CI speech performance, it can be difficult to separate within-subject training effects from across-subject variability. To avoid this, a within-subject control procedure was adopted instead of a separate control group. The within-subject control procedure also allowed “perceptual learning” effects to be better separated from ‘‘procedural learning’’ effects (i.e., task familiarization or test experience).

All pre-training, post-training and follow-up tests were performed in a soundproof booth that was regularly used for patient evaluations and that CI participants were familiar with. Testing was conducted by a researcher who was unknown to participants before the study started. Only the participant and the researcher were present in the room.

Vowel and consonant stimuli were digitized natural productions (44.1 kHz sampling rate, 16-bit resolution) from two adult, native French-speaking talkers (one man and one woman) that were not included in the training stimuli. Vowels included 10 tokens presented without context (vowel only), for a total of 20 stimuli in the stimulus set. Consonants included 19 tokens presented in a /a/-consonant-/a/ context, for a total of 38 stimuli in the stimulus set. Vowel recognition in quiet was measured using a 10-alternative, forced-choice (10AFC) closed-set paradigm. Consonant recognition in quiet was measured using a 19AFC closed-set paradigm. Vowel and consonant stimuli were presented over a single loudspeaker at 65 dBA, with the listener seated facing the loudspeaker 1 m away. During testing, one stimuli was randomly selected from the stimulus set (without replacement) and presented to the listener, who responded by clicking on one of the response choices shown on a computer screen (see Fig 2 for the vowel and consonant response screens). After selecting the response, the next token was presented. For each test run, the stimulus set was presented once. No feedback was provided, and listeners were instructed to guess if they were unsure of the correct answer. Three to four test runs were performed for each participant. Data were averaged across all test runs.

**Fig 2. Response screens showing the stimuli used for the vowel (left) and consonant recognition tests (right) in quiet.**

Speech recognition thresholds (SRTs), defined as the signal-to-noise ratio (SNR) that produces 50% correct sentence recognition in steady noise [23], were measured using French Hearing-in-noise-test (HINT) sentences [24] produced by the same male and female talkers that produced the vowel and consonant test stimuli. Note that the sentences were novel stimuli recorded for this study (44.1 kHz sampling rate, 16-bit resolution). White noise was filtered to match the combined long-term spectrum across all test sentences. SRTs were adaptively measured using an open-set paradigm. Sentences and noise were presented from a single loudspeaker; the listener was seated facing the loudspeaker 1 m away. The onset and offset of the noise were 500 ms before and after the sentence, respectively. The level of the sentence was fixed at 65 dBA, and the noise level was adapted. The stimulus set consisted of 100 sentences. During testing, a sentence was randomly selected from the stimulus set (without replacement) and presented at the designated SNR. The listener responded by repeating as many words as heard, and the experimenter marked the correct responses. The SNR was adjusted according to the correctness of response, using “Rule 3” from Chan et al. [25]: if 50% or more of the words were repeated correctly, the SNR was reduced; if not, the SNR was increased. If the SNR exceeded 25 dB during a test run, the run was aborted. The initial SNR was 10 dB. The initial step size was 4 dB for the first two reversals in SNR, and the final step size was 2 dB. The first sentence was played until the listener correctly repeated 50% of the words. If the listener failed to repeat 50% of the words, the sentence was played again and the SNR was increased (in 4 dB steps) until 50% of the words were repeated correctly. If the SNR exceeded 25 dB SNR, the test run was aborted and a new test run was begun with a new initial sentence. After correctly repeating 50% of the words in the first sentence, the remaining 19 sentences in the list were presented one time. The final six reversals in SNR were averaged to calculate the SRT for the test run. Three test runs were performed for each listening condition (CI-only for unilateral CI and bimodal patients; binaural for bilateral CI and bimodal patients) and data were averaged across all three test runs.

Phoneme recognition and SRTs were also measured in 16 NH listeners. For unilateral CI patients, vowel recognition was measured first, followed by consonant recognition and SRTs in noise. For bilateral CI patients, binaural vowel recognition was measured first to familiarize patients with the task using their everyday listening condition. Next, vowel recognition was measured in each CI ear alone to determine the poorer ear that would be used for training. Next, binaural consonant recognition and SRTs were measured; due to time constraints, consonant recognition and SRTs were measured only with binaural listening. For bimodal CI patients, binaural vowel recognition was measured first to familiarize patients with the task using their everyday listening condition, followed by CI-only vowel recognition. Next, binaural consonant recognition was measured, followed by CI-only consonant recognition. Next, binaural SRTs were measured, followed by CI-only SRTs. For CI-only performance, the contralateral hearing aid was removed, and the ear was plugged.

**Training protocol**

Training was performed using the recently developed French version of AngelSound, which is similar in structure to the previous English and Mandarin versions of AngelSound. The French AngelSound stimuli are high-quality recordings (44.1 kHz sampling rate, 16-bit resolution) of French phonemes, words and sentences produced by multiple native French talkers. For the phonemic contrast training used in the present study, there are more than 1000 French monosyllable words produced by 2 male and 2 female native French talkers that were not used for testing. All vowel and consonant stimuli were tagged with relevant acoustic features that allow for adjusting the degree of training difficulty to include relatively strong or weak acoustic contrasts. For the vowel stimuli, acoustic features included first formant (high, middle, or low), second formant (front or back), third formant (rounded or unrounded), and nasality (absent or present). For the consonant stimuli, acoustic features included voicing (voiced or unvoiced), manner (stop, nasal, fricative, lateral approximate, or semi-vowel), and place of articulation (front, middle, or back).

After completing baseline measures, participants were provided with a study-specific version of French AngelSound via web link or on a USB thumb drive; the software allowed for only phonemic contrast training, and all other training modules were disabled. Participants were extensively instructed how to install the software and how to perform the training. None reported any difficulty installing or using the training software.

The training protocol was similar to that used in previous studies by Fu and colleagues [5,6,7,8,11,12,15,26]. Participants were asked to train for 30 minutes per day, 5 days per week for 1 month, resulting in 10 hours of total training. The training software logged all time spent training. After completing 1 month of home training, participants returned to the lab and vowel recognition, consonant recognition, and SRTs were re-measured; participants also completed the SSQ-12 questionnaire. After completing these post-training outcome measures, training was stopped. One month after training was stopped, participants returned to the lab for follow-up measures to observe whether any post-training had been retained.

Pre-training vowel and consonant scores were used to suggest the appropriate level of difficulty. This was recommended as the most appropriate training level, but participants were free to train on any level of difficulty that they desired. According to participant interviews, the majority of patients trained with all levels, typically using the recommended levels in the beginning and increasing the difficulty when they wanted to diversify their training and decreasing it if they found it too difficult. Within each level of difficulty, there were multiple sublevels of difficulty in which the phonemic contrasts were reduced. Levels of difficulty included:

- Level 1 (Easy): Simple discrimination of phonemes in words. In each trial, three sounds were played in sequence; two of the sounds were identical and the third was different. The three sounds were randomly assigned to three intervals (labeled “Son 1,” “Son 2”, Son 3”), and participants were asked to choose which sound was different. Visual feedback was provided as to the correctness of response. If the participant did not respond correctly, auditory and visual feedback were provided in which the three sounds, now labeled with the appropriate words (e.g., “gare” [gaʁ], “guerre” [gɛʁ], “toque” [tɔk], and “tek” [tɛk] were replayed in the same sequence, allowing the participant to compare their response to the correct response. Within Level 1, sublevels of difficulty were increased by reducing the phonemic contrast across response options.
- Level 2 (Easy to Medium): Identification of phonemes in words with audio-visual preview. In each trial, two stimuli were shown onscreen (with the appropriate word labels) and played in sequence. One of the two stimuli was then played using a different talker than for the preview, and participants clicked on the correct word. Only one phoneme differed between response choices (e.g., “toque”, “tek”). Visual feedback was provided as to the correctness of response. If the participant did not respond correctly, auditory and visual feedback were provided in which the two stimuli, produced by the different talker and labeled with the appropriate words (e.g., “toque”, “tek”), were replayed, allowing the participant to compare their response to the correct response. Within Level 2, sublevels of difficulty were increased by reducing the phonemic contrast across response options.
- Level 3 (Medium): Identification of phonemes in words without preview. In each trial, two response choices were shown onscreen, with only one phoneme differing between response choices (e.g., “toque”, “tek”). A stimulus was played that corresponds to one of the response choices, and participants clicked on the response choice that best matched the stimulus. Visual feedback was provided as to the correctness of response. If the participant did not respond correctly, auditory and visual feedback were provided in which stimuli corresponding to the two response choices were played back, allowing the participant to compare their response to the correct response. Within Level 3, sublevels of difficulty were increased by reducing the phonemic contrast across response choices and by increasing the number of response choices.
- Level 4 (Medium to Difficult): Identification of phonemes in words without preview. Level 4 was similar to Level 3, except that the sublevels of difficulty were increased according to perceptual confusions (rather than acoustic contrasts) and by increasing the number of response choices.
- Level 5 (Difficult). Identification of phonemes in words in multi-talker babble without feedback. Level 5 was similar to Level 3, except that phoneme identification was measured in the presence of multi-talker speech babble. The number of response choices was fixed at 4. For each training run, identification was trained at a fixed SNR. A stimulus was played that corresponded to one of the response choices, and participants clicked on the response choice that best matched the stimulus. Visual feedback was provided as to the correctness of response. If the participant did not respond correctly, auditory and visual feedback were provided in which stimuli were played back at the fixed SNR, allowing the participant to compare their response to the correct response. Within Level 5, sublevels of difficulty were increased by reducing the SNR.

Participants were asked to train with the vowel and consonant stimuli. Participants were instructed to train in a quiet environment with the computer speaker output set to a comfortably loud level. Unilateral and bimodal CI patients were instructed to train with the CI ear only; bilateral CI patients were instructed to train with the poorer ear only (identified from baseline testing results), or with the more recently implanted ear if performance was similar across ears. Participants were instructed to train for 30 minutes per day, 5 days per week, for 1 month (10 hours of training in total). Participants were cautioned against doing too little or too much training in the 1-month training period. Participants were also instructed that they could break up each day’s training into multiple session (e.g., 3 sessions of 10 minutes each) to accommodate their schedule, attentiveness, fatigue, etc. Each training run consisted of 25 stimuli and took 5-10 minutes to complete. Results were recorded only when a training run was completed. After completing the home training, training data were shared with the experimenter, including the total time spent training, the levels of difficulty trained, and the percent correct for each training run.

**Results**

**Phoneme recognition and SRTs in NH listeners**

Vowel and consonant recognition in quiet were measured in 16 NH listeners, and SRTs in noise were measured in 13 NH listeners. Mean vowel recognition was 99.4 ± 2.0 percent correct, mean consonant recognition was 99.5±1.4 percent correct, and mean SRTs were -5.3 ± 2.2 dB.

**Pre-training, post-training, and follow-up measures in CI listeners**

**Vowel recognition in quiet**

Fig 3 shows pre-training, post-training, and follow-up vowel recognition scores for individual CI listeners as a function of test run. Participants completed 3-4 test runs. A series of linear mixed model (LMM) analyses were performed on data from unilateral, bilateral (poorer CI-only and binaural listening), and bimodal groups (CI-only and binaural listening). For all analyses, test (Pre, Post, Follow-up) and test run (1, 2, 3, 4) were fixed factors, and participant was a random factor. Complete results are shown in Table 2. For all groups and listening conditions, a significant effect was observed for test (p < 0.001), but not for test run; there were no significant interactions.

**Fig 3. Vowel recognition scores for individual CI listeners as a function of test run.** Pre-training (left column), post-training (middle column), and follow-up data (right column) are shown for the unilateral group (top row), bilateral group with poorer CI-only and binaural listening (middle two rows), and the bimodal group with CI-only and binaural listening (bottom two rows).

**Table 2. Results from LMM analyses of vowel test run data.** Results are shown for the unilateral, bilateral, and bimodal groups with CI-only and binaural listening. Significant effects are indicated by italics and asterisks. Significant post-hoc pairwise comparisons (p < 0.05) are shown after Bonferroni adjustment.

|  |  | **CI-only** | | | | | **Binaural** | | | | |
| --- | --- | --- | --- | --- | --- | --- | --- | --- | --- | --- | --- |
| **Vowels** |  | **dF, res** | **F** | **p** | **post-hoc** | **dF, res** | | **F** | **p** | **post-hoc** |  |
| Unilateral | Test | 2, 47 | 23.0 | *<0.001** | Pre < Post, Follow-up |  | |  |  |  |  |
|  | Run | 3, 47 | 2.7 | 0.059 |  |  | |  |  |  |  |
|  | Test x Run | 6, 47 | 0.9 | 0.538 |  |  | |  |  |  |  |
| Bilateral | Test | 2, 41 | 31.2 | *<0.001** | Pre < Post, Follow-up | 2, 41 | | 33.8 | *<0.001** | Pre < Post, Follow-up |  |
|  | Run | 3, 41 | 2.0 | 0.134 |  | 3, 41 | | 0.9 | 0.471 |  |  |
|  | Test x Run | 5, 41 | 1.9 | 0.120 |  | 5, 41 | | 0.2 | 0.923 |  |  |
| Bimodal | Test | 2, 41 | 24.9 | *<0.001** | Pre < Post, Follow-up | 2, 41 | | 35.0 | *<0.001** | Pre < Post, Follow-up |  |
|  | Run | 3, 41 | 1.6 | 0.212 |  | 3, 41 | | 0.9 | 0.428 |  |  |
|  | Test x Run | 5, 41 | 0.8 | 0.566 |  | 5, 41 | | 1.0 | 0.409 |  |  |

The top panels of Fig 4 show mean pre-training, post-training, and follow-up vowel recognition scores with CI-only and binaural listening; mean scores are also shown at the top of Table 3. LMM analyses were performed on the vowel recognition data with CI-only listening [fixed factors: test (Pre, Post, Follow-up) and group (UNI, BI, BM); random factor: participant] and with binaural listening [fixed factors: test (Pre, Post, Follow-up) and group (BI, BM); random factor: participant]. Complete results are shown at the top of Table 4. For both CI-only and binaural listening, pre-training scores were lower than post-training (p < 0.001) and follow-up scores (p < 0.001), with no significant difference between post-training and follow-up scores.

The bottom panels of Fig 4 show mean improvement in vowel recognition with CI-only and binaural listening at post-training and follow-up, relative to pre-training scores; mean improvement data are shown at the bottom of Table 3. Across the unilateral, bilateral, and bimodal CI groups, mean CI-only post-training scores were 19.1 percentage points better than pre-training scores; mean follow-up scores remained 21.5 percentage points better than pre-training scores. Across bilateral and bimodal CI groups, mean binaural post-training scores were 20.5 percentage points better than pre-training scores; mean follow-up scores remained 19.8 percentage points better than pre-training scores. LMM analyses were performed on the training benefit data with CI-only listening [fixed factors: test (Post, Follow-up) and group (UNI, BI, BM); random factor: participant] and with binaural listening [fixed factors: test (Post, Follow-up) and group (BI, BM); random factor: participant]. Complete results are shown at the bottom of Table 4. There were no significant effects of test or group on training benefit with CI-only or binaural listening.

**Fig 4. Mean vowel recognition and training benefit.** The top panels show vowel recognition scores with CI-only (left) and binaural listening (right); the dashed lines show mean NH performance. The bottom panels show training benefit with CI-only (left) and binaural listening (right). The error bars show the standard deviation. The asterisks show significantly different scores.

**Table 3. Mean vowel recognition scores and training benefit.** Data are shown for the different listening groups with CI-only and bimodal listening. Training benefit for post-training and follow-up were calculated relative to pre-training scores.

|  |  | **Pre-training** | | **Post-training** | | **Follow-up** | |
| --- | --- | --- | --- | --- | --- | --- | --- |
|  | **Vowels**  **(percent correct)** | **Mean** | **STD** | **Mean** | **STD** | **Mean** | **STD** |
| CI-only | Unilateral | 58.3 | 20.2 | 75.3 | 14.5 | 81.0 | 12.3 |
|  | Bilateral | 51.3 | 16.5 | 71.7 | 13.5 | 71.8 | 14.7 |
|  | Bimodal | 40.7 | 13.5 | 50.1 | 8.9 | 62.0 | 15.1 |
|  | AVE | 50.1 | 17.4 | 69.2 | 15.6 | 71.6 | 15.3 |
| Binaural | Bilateral | 63.0 | 24.0 | 80.6 | 13.8 | 81.7 | 11.5 |
|  | Bimodal | 49.0 | 12.1 | 72.3 | 15.8 | 69.9 | 15.6 |
|  | AVE | 56.0 | 19.4 | 76.5 | 14.7 | 75.8 | 14.3 |
|  |  |  |  |  |  |  |  |
|  | **Training benefit**  **(percentage points)** |  |  | **Mean** | **STD** | **Mean** | **STD** |
| CI-only | Unilateral |  |  | 17.1 | 7.9 | 22.8 | 18.2 |
|  | Bilateral |  |  | 20.4 | 7.2 | 20.5 | 10.3 |
|  | Bimodal |  |  | 19.8 | 9.2 | 21.3 | 16.2 |
|  | AVE |  |  | 19.1 | 7.7 | 21.5 | 14.2 |
| Binaural | Bilateral |  |  | 17.6 | 10.7 | 18.7 | 14.0 |
|  | Bimodal |  |  | 23.3 | 14.1 | 20.9 | 11.7 |
|  | AVE |  |  | 20.5 | 12.2 | 19.8 | 12.2 |

**Table 4. Results from LMM analyses of vowel recognition and training benefit data.** Results are shown for CI-only and binaural listening. Significant post-hoc pairwise comparisons (p < 0.05) are shown after Bonferroni adjustment.

|  |  |  | **dF, res** | | **F** | | **p** | | **post-hoc** | |
| --- | --- | --- | --- | --- | --- | --- | --- | --- | --- | --- |
| Vowel recognition | CI-only | Test | 2, 30 | 36.2 | | *<0.001** | | Pre < Post, Follow-up | |  |
|  |  | Group | 2, 15 | 2.4 | | 0.122 | |  | |  |
|  |  | Test x Group | 4, 30 | 0.2 | | 0.944 | |  | |  |
|  | Binaural | Test | 2, 20 | 27.6 | | *<0.001** | | Pre < Post, Follow-up | |  |
|  |  | Group | 1, 10 | 1.8 | | 0.207 | |  | |  |
|  |  | Test x Group | 2, 20 | 0.4 | | 0.655 | |  | |  |
| Training benefit | CI-only | Test | 1, 15 | 0.8 | | 0.376 | |  | |  |
|  |  | Group | 2, 15 | 0.1 | | 0.993 | |  | |  |
|  |  | Test x Group | 2, 15 | 0.4 | | 0.678 | |  | |  |
|  | Binaural | Test | 1, 10 | 0.1 | | 0.729 | |  | |  |
|  |  | Group | 1, 10 | 0.3 | | 0.577 | |  | |  |
|  |  | Test x Group | 1, 10 | 0.9 | | 0.378 | |  | |  |

**Consonant recognition in quiet**

Fig 5 shows pre-training, post-training, and follow-up consonant recognition scores for individual CI listeners as a function of test run. Note that CI-only performance was not measured in bilateral CI users due to time constraints. Participants completed 2-3 test runs. A series of LMM analyses were performed on consonant test run data from unilateral, bilateral (binaural listening), and bimodal groups (CI-only and binaural listening). For all analyses, test (Pre, Post, Follow-up) and test run (1, 2, 3) were fixed factors, and participant was a random factor. Complete results are shown in Table 5. For all groups and listening conditions, a significant effect was observed for test (p < 0.001), There was a significant effect of test run only for the unilateral group, where run 1 was significantly lower than run 3 (p = 0.028), with no significant differences between runs 1 and 2 or between runs 2 and 3.

**Fig 5. Consonant recognition scores for individual CI listeners as a function of test run.** Pre-training (left column), post-training (middle column), and follow-up data (right column) are shown for the unilateral group (top row), bilateral group with binaural listening (middle row), and the bimodal group with CI-only and binaural listening (bottom two rows).

**Table 5. Results from LMM analyses of consonant test run data.** Results are shown for the unilateral, bilateral, and bimodal groups with CI-only and binaural listening. Significant effects are indicated by italics and asterisks. Significant post-hoc pairwise comparisons (p < 0.05) are shown after Bonferroni adjustment.

|  |  | **CI-only** | | | | **Binaural** | | | |
| --- | --- | --- | --- | --- | --- | --- | --- | --- | --- |
| **Consonants** |  | **dF, res** | **F** | **p** | **post-hoc** | **dF, res** | **F** | **p** | **post-hoc** |
| Unilateral | Test | 2, 37 | 40.4 | *<0.001** | Pre < Post, Follow-up |  |  |  |  |
|  | Run | 2, 37 | 3.9 | 0.029* | R1 < R3 |  |  |  |  |
|  | Test x Run | 4, 37 | 0.7 | 0.580 |  |  |  |  |  |
| Bilateral | Test |  |  |  |  | 2, 40 | 18.1 | *<0.001** | Pre < Post, Follow-up |
|  | Run |  |  |  |  | 2, 40 | 1.3 | 0.272 |  |
|  | Test x Run |  |  |  |  | 4, 40 | 0.2 | 0.961 |  |
| Bimodal | Test | 2, 35 | 25.1 | *<0.001** | Pre < Post, Follow-up | 2, 37 | 20.4 | *<0.001** | Pre < Post, Follow-up |
|  | Run | 2, 35 | 0.1 | 0.938 |  | 2, 37 | 1.1 | 0.381 |  |
|  | Test x Run | 4, 35 | 0.4 | 0.777 |  | 4, 37 | 0.4 | 0.784 |  |

The top panels of Fig 6 show mean pre-training, post-training, and follow-up consonant recognition scores with CI-only and binaural listening; mean scores are also shown at the top of Table 6. LMM analyses were performed on the consonant recognition data with CI-only listening [fixed factors: test (Pre, Post, Follow-up) and group (UNI, BM); random factor: participant] and with binaural listening [fixed factors: test (Pre, Post, Follow-up) and group (BI, BM); random factor: participant]. Complete results are shown at the top of Table 6. For both CI-only and binaural listening, pre-training scores were lower than post-training (p < 0.001) and follow-up scores (p < 0.001), with no significant difference between post-training and follow-up scores. CI-only consonant recognition was also significantly poorer for the bimodal than for the unilateral group (p = 0.042); there were no significant differences among the remaining groups.

The bottom panels of Fig 6 show mean improvement in consonant recognition with CI-only and binaural listening at post-training and follow-up, relative to pre-training scores; mean improvement data are shown at the bottom of Table 6. Across the unilateral and bimodal CI groups, mean CI-only post-training scores were 15.9 percentage points better than pre-training scores; mean follow-up scores remained 12.9 percentage points better than pre-training scores. Across bilateral and bimodal CI groups, mean binaural post-training scores were 17.3 percentage points better than pre-training scores; mean follow-up scores remained 13.1 percentage points better than pre-training scores. LMM analyses were performed on the training benefit data with CI-only listening [fixed factors: test (Post, Follow-up) and group (UNI, BM); random factor: participant] and with binaural listening [fixed factors: test (Post, Follow-up) and group (BI, BM); random factor: participant]. Complete results are shown at the bottom of Table 7. There were no significant effects of test or group on training benefit with CI-only or binaural listening.

**Fig 6. Mean consonant recognition and training benefit.** The top panels show consonant recognition scores with CI-only (left) and binaural listening (right); the dashed lines show mean NH performance. The bottom panels show training benefit with CI-only (left) and binaural listening (right). The error bars show the standard deviation. The asterisks show significantly different scores.

**Table 6. Mean consonant recognition scores and training benefit.** Data are shown for the different listening groups with CI-only and bimodal listening. Training benefit for post-training and follow-up were calculated relative to pre-training scores.

|  |  | **Pre-training** | | **Post-training** | | **Follow-up** | |
| --- | --- | --- | --- | --- | --- | --- | --- |
|  | **Consonants**  **(percent correct)** | **Mean** | **STD** | **Mean** | **STD** | **Mean** | **STD** |
| CI-only | Unilateral | 68.2 | 17.0 | 82.9 | 11.6 | 85.4 | 10.9 |
|  | Bimodal | 45.2 | 26.1 | 62.3 | 17.2 | 62.5 | 19.2 |
|  | AVE | 56.7 | 24.0 | 72.6 | 17.6 | 74.0 | 19.0 |
| Binaural | Bilateral | 74.1 | 20.6 | 86.0 | 12.0 | 83.5 | 15.0 |
|  | Bimodal | 52.4 | 23.7 | 66.3 | 15.7 | 69.1 | 17.3 |
|  | AVE | 63.3 | 23.8 | 76.1 | 16.8 | 76.3 | 17.1 |
|  |  |  |  |  |  |  |  |
|  | **Training benefit (percentage points)** |  |  | **Mean** | **STD** | **Mean** | **STD** |
| CI-only | Unilateral |  |  | 14.7 | 10.3 | 11.9 | 10.1 |
|  | Bimodal |  |  | 17.1 | 13.5 | 13.9 | 13.1 |
|  | AVE |  |  | 15.9 | 11.4 | 12.9 | 11.1 |
| Binaural | Bilateral |  |  | 17.2 | 7.3 | 9.4 | 7.7 |
|  | Bimodal |  |  | 17.3 | 12.5 | 16.7 | 14.0 |
|  | AVE |  |  | 17.3 | 9.6 | 13.1 | 11.3 |

**Table 7. Results from LMM analyses of consonant recognition and training benefit data.** Results are shown for CI-only and binaural listening. Significant post-hoc pairwise comparisons (p < 0.05) are shown after Bonferroni adjustment.

|  |  |  | **dF, res** | **F** | **p** | **post-hoc** |
| --- | --- | --- | --- | --- | --- | --- |
| Consonant recognition | CI-only | Test | 2, 20 | 25.0 | *<0.001** | Pre < Post, Follow-up |
|  |  | Group | 1, 10 | 5.4 | *0.042** | Bimodal < unilateral |
|  |  | Test x Group | 2, 20 | 0.1 | 0.889 |  |
|  | Binaural | Test | 2, 20 | 14.7 | *<0.001** | Pre < Post, Follow-up |
|  |  | Group | 1, 10 | 3.7 | 0.082 |  |
|  |  | Test x Group | 2, 20 | 0.9 | 0.433 |  |
| Training  benefit | CI-only | Test | 1, 10 | 0.9 | 0.363 |  |
|  |  | Group | 1, 10 | 0.1 | 0.842 |  |
|  |  | Test x Group | 1, 10 | 0.6 | 0.471 |  |
|  | Binaural | Test | 1, 10 | 0.1 | 0.886 |  |
|  |  | Group | 1, 10 | 0.5 | 0.489 |  |
|  |  | Test x Group | 1, 10 | 3.8 | 0.810 |  |

Training benefits for CI-only and binaural listening was also compared between vowel and consonant recognition. LMM analyses were performed on the training benefit data with CI-only listening [fixed factors: phoneme (vowel, consonant), test (Post, Follow-up) and group (UNI, BM); random factor: participant] and with binaural listening [fixed factors: test (Post, Follow-up) and group (BI, BM); random factor: participant]. With CI-only listening, there were no significant effects of phoneme, test, or group on training benefits. With binaural listening, training benefits were significantly larger for vowels than for consonants [F(1,30) = 12.1, p = 0.002]. There were no significant effects of test or group, and no significant interactions.

**Sentence recognition in noise**

Fig 7 shows pre-training, post-training, and follow-up SRTs for individual CI listeners as a function of test run. Note that CI-only performance was not measured in bilateral CI users due to time constraints. Participants completed 2-3 test runs. A series of LMM analyses were performed on SRT test run data from unilateral, bilateral (binaural listening), and bimodal groups (CI-only and binaural listening). For all analyses, test (Pre, Post, Follow-up) and test run (1, 2, 3) were fixed factors, and participant was a random factor; note that SRTs > 25 dB were excluded from the analyses. Complete results are shown in Table 8. For all groups and listening conditions, a significant effect was observed for test (p < 0.001), There was a significant effect of test run only for the unilateral group, where run 1 was significantly lower than run 3 (p = 0.028), with no significant differences between runs 1 and 2 or between runs 2 and 3.

**Fig 7. SRTs for individual CI listeners as a function of test run.** Pre-training (left column), post-training (middle column), and follow-up data (right column) are shown for the unilateral group (top row), bilateral group with binaural listening (middle row), and the bimodal group with CI-only and binaural listening (bottom two rows). The horizontal line shows the maximum SRT (25 dB) before the test run was aborted. The open symbols show test runs that exceeded the maximum SRT (25 dB SNR),

**Table 8. Results from LMM analyses of SRT test run data.** Results are shown for the unilateral, bilateral, and bimodal groups with CI-only and binaural listening. Significant effects are indicated by italics and asterisks. Significant post-hoc pairwise comparisons (p < 0.05) are shown after Bonferroni adjustment.

|  |  | **CI-only** | | | | **Binaural** | | | |
| --- | --- | --- | --- | --- | --- | --- | --- | --- | --- |
| **SRTs** |  | **dF, res** | **F** | **p** | **post-hoc** | **dF, res** | **F** | **p** | **post-hoc** |
| Unilateral | Test | 2, 35 | 15.8 | *<0.001** | Pre > Post, Follow-up |  |  |  |  |
|  | Run | 2, 35 | 4.3 | 0.021* | R1 > R3 |  |  |  |  |
|  | Test x Run | 4, 35 | 1.0 | 0.408 |  |  |  |  |  |
| Bilateral | Test |  |  |  |  | 2, 38 | 13.1 | *<0.001** | Pre > Post, Follow-up |
|  | Run |  |  |  |  | 2, 38 | 9.0 | *<0.001** |  |
|  | Test x Run |  |  |  |  | 2, 38 | 0.2 | 0.943 |  |
| Bimodal | Test | 2, 33 | 30.2 | *<0.001** | Pre > Post, Follow-up | 2, 36 | 37.1 | *<0.001** | Pre > Post, Follow-up |
|  | Run | 2, 33 | 0.6 | 0.544 |  | 2, 36 | 2.0 | 0.149 |  |
|  | Test x Run | 4, 33 | 1.6 | 0.198 |  | 2, 36 | 1.5 | 0.210 |  |

The top panels of Fig 8 show mean pre-training, post-training, and follow-up SRTs with CI-only and binaural listening; mean scores are also shown at the top of Table 9. Note that only SRTs <25 dB SNR were included in the figures and analyses. For example, when CI-only listening SRTs <25 dB SNR could not be obtained, LMM analyses were performed on the SRT data with CI-only listening [fixed factors: test (Pre, Post, Follow-up) and group (UNI, BM); random factor: participant] and with binaural listening [fixed factors: test (Pre, Post, Follow-up) and group (BI, BM); random factor: participant]. Complete results are shown at the top of Table 10. For both CI-only and binaural listening, pre-training SRTs were lower than post-training (p < 0.001) and follow-up SRTs (p < 0.001), with no significant difference between post-training and follow-up scores. There was no significant effect of group.

The bottom panels of Fig 8 show mean improvement in SRTs with CI-only and binaural listening at post-training and follow-up, relative to pre-training scores; mean improvement data are shown at the bottom of Table 9. Across the unilateral and bimodal CI groups, mean CI-only post-training SRTs were 6.5 dB better than pre-training SRTs; mean follow-up SRTs remained 7.6 dB better than pre-training SRTs. Across bilateral and bimodal CI groups, mean binaural post-training SRTs were 6.2 dB better than pre-training SRTs; mean follow-up scores remained 7.1 dB better than pre-training SRTs. LMM analyses were performed on the training benefit data with CI-only listening [fixed factors: test (Post, Follow-up) and group (unilateral, bimodal); random factor: participant] and with binaural listening [fixed factors: test (Post, Follow-up) and group (bilateral, bimodal); random factor: participant]. Complete results are shown at the bottom of Table 10. There were no significant effects of test or group on training benefit with CI-only or binaural listening.

**Fig 8. Mean SRTs and training benefit.** The top panels show SRTs with CI-only (left) and binaural listening (right); the dashed lines show mean NH performance. The bottom panels show training benefit with CI-only (left) and binaural listening (right). The error bars show the standard deviation. The asterisks show significantly different scores. Note that only SRTs <25 dB were included.

**Table 9. Mean SRTs and training benefit.** Data are shown for the different listening groups with CI-only and bimodal listening. Training benefit for post-training and follow-up were calculated relative to pre-training scores. Note that only SRTs <25 dB were included in the calculations.

|  |  | **Pre-training** | | **Post-training** | | **Follow-up** | |
| --- | --- | --- | --- | --- | --- | --- | --- |
|  | **SRT (dB SNR)** | **Mean** | **STD** | **Mean** | **STD** | **Mean** | **STD** |
| CI-only | Unilateral | 9.7 | 4.6 | 5.0 | 3.4 | 3.1 | 3.6 |
|  | Bimodal | 12.0 | 5.7 | 10.8 | 8.1 | 8.0 | 7.8 |
|  | AVE | 10.9 | 4.8 | 7.9 | 6.6 | 5.6 | 6.3 |
| Binaural | Bilateral | 9.6 | 7.2 | 4.0 | 4.2 | 3.8 | 4.0 |
|  | Bimodal | 11.7 | 5.5 | 7.0 | 5.4 | 4.5 | 3.2 |
|  | AVE | 10.5 | 6.2 | 5.5 | 4.9 | 4.1 | 3.5 |
|  |  |  |  |  |  |  |  |
|  | **Training benefit (dB)** |  |  | **Mean** | **STD** | **Mean** | **STD** |
| CI-only | Unilateral |  |  | 5.9 | 2.1 | 6.1 | 0.1 |
|  | Bimodal |  |  | 7.0 | 5.9 | 9.1 | 3.6 |
|  | AVE |  |  | 6.5 | 4.0 | 7.6 | 2.8 |
| Binaural | Bilateral |  |  | 5.7 | 3.3 | 5.9 | 5.3 |
|  | Bimodal |  |  | 6.9 | 3.8 | 8.5 | 4.9 |
|  | AVE |  |  | 6.2 | 3.4 | 7.1 | 5.0 |

**Table 10. Results from LMM analyses of SRTs and training benefit data.** Results are shown for CI-only and binaural listening. Significant post-hoc pairwise comparisons (p < 0.05) are shown after Bonferroni adjustment.

|  |  |  | **dF, res** | **F** | **p** | **post-hoc** |
| --- | --- | --- | --- | --- | --- | --- |
| SRT | CI-only | Test | 2, 16 | 27.2 | *<0.001** | Pre > Post, Follow-up |
|  |  | Group | 1, 10 | 2.9 | 0.117 |  |
|  |  | Test x Group | 2, 16 | 0.5 | 0.609 |  |
|  | Binaural | Test | 2, 19 | 24.8 | *<0.001** | Pre > Post, Follow-up |
|  |  | Group | 1, 10 | 0.7 | 0.408 |  |
|  |  | Test x Group | 2, 19 | 1.3 | 0.301 |  |
| Training benefit | CI-only | Test | 1, 6 | 1.1 | 0.335 |  |
|  |  | Group | 1, 7 | 1.2 | 0.313 |  |
|  |  | Test x Group | 1, 6 | 2.7 | 0.152 |  |
|  | Binaural | Test | 1, 9 | 2.2 | 0.176 |  |
|  |  | Group | 1, 9 | 0.6 | 0.454 |  |
|  |  | Test x Group | 1, 9 | 1.2 | 0.294 |  |

**Compliance with the testing and training protocol**

The mean amount of training completed was 10.3±1.7 hours, or 31.7±4.0 minutes per day. All participants were tested in the lab. The amount of training completed was compared to the improvements in post-training and follow-up performance with CI-only and binaural listening. Pearson correlations showed no significant relationship between training benefit and the amount of training completed for any of the outcome measures.

**Speech, spatial, and quality of hearing questionnaire**

Fig 9 shows mean pre-training, post-training, and follow-up SSQ scores for the speech, spatial, and quality subsections, as well as the total SSQ score; mean data are shown in Table 11. LMM analysis was performed on the SSQ data, with group (unilateral, bilateral, bimodal), subsection (speech, spatial, quality) and test (Pre, Post, Follow-up) as fixed factors and participant as the random factor. Results showed significant effects for group [F(2, 15) = 4.1, p = 0.038] and subsection [F(2, 120) = 27.6, p < 0.001], but not for test [F(2, 120) = 0.2, p = 0.883]; there were no significant interactions. While the effect of group was significant, post-hoc Bonferroni-corrected pairwise comparisons showed no significant differences among groups. Bonferroni-corrected pairwise comparisons showed that SSQ scores were significantly higher for the quality subsection than for the speech (p < 0.001) and spatial subsections (p < 0.001), with no significant difference between the speech and spatial subsections. LMM analysis were performed on the SSQ total score data, with group (unilateral, bilateral, bimodal) and test (Pre, Post, Follow-up) as fixed factors and participant as the random factor. Results showed significant effects for group [F(2, 15) = 4.0, p = 0.031], but not for test [F(2, 30) = 0.2, p = 0.825]; there were no significant interactions. While the effect of group was significant, post-hoc Bonferroni-corrected pairwise comparisons showed no significant differences among groups.

**Fig 9. Mean pre-training, post-training, and follow-up SSQ scores for the different CI groups** Data are shown for the speech, (top left), spatial (top right), and quality subsections (bottom left), and for the total score (bottom right). In all panels, the error bars show the standard deviation.

**Table 11. Mean vowel pre-train, post-train, and follow-up SSQ scores.** Data are shown for the speech, spatial, and quality subsections, and for total score, for the different listening groups.

|  |  | **Pre-training** | | **Post-training** | | **Follow-up** | |
| --- | --- | --- | --- | --- | --- | --- | --- |
|  | **SSQ** | **Mean** | **STD** | **Mean** | **STD** | **Mean** | **STD** |
| Speech | Unilateral | 3.5 | 1.6 | 3.2 | 1.1 | 3.1 | 1.3 |
|  | Bilateral | 5.5 | 1.3 | 5.2 | 1.6 | 5.8 | 1.8 |
|  | Bimodal | 3.0 | 1.5 | 2.9 | 1.9 | 2.5 | 2.2 |
| Spatial | Unilateral | 3.3 | 1.3 | 3.2 | 2.1 | 3.1 | 2.5 |
|  | Bilateral | 4.8 | 1.8 | 5.8 | 2.6 | 5.6 | 2.7 |
|  | Bimodal | 2.9 | 2.2 | 2.8 | 1.3 | 2.2 | 2.1 |
| Quality | Unilateral | 4.1 | 1.1 | 4.6 | 1.6 | 4.0 | 1.2 |
|  | Bilateral | 6.3 | 1.1 | 6.4 | 1.1 | 6.6 | 1.4 |
|  | Bimodal | 4.9 | 1.9 | 4.4 | 2.9 | 4.7 | 3.0 |
| Total | Unilateral | 3.7 | 1.3 | 3.7 | 1.5 | 3.4 | 1.5 |
|  | Bilateral | 5.6 | 1.4 | 5.9 | 1.6 | 6.0 | 1.6 |
|  | Bimodal | 3.7 | 1.6 | 3.4 | 1.9 | 3.2 | 2.4 |

**Discussion**

The computer-based home training showed significant benefits for phoneme recognition in quiet and sentence recognition in noise for the present French CI patients, consistent with previous home-training studies with English-, German- and Mandarin-speaking CI patients [5,6,7,8,9,10,11,12,14,15]. The phonemic contrast training appeared to generalize to SRTs in noise, consistent with previous studies [6,7,14]. For bilateral and bimodal CI patients, training with a single CI ear generalized to improved binaural performance, consistent with Zhang et al. [12]. For all CI groups and listening conditions, follow-up measures were not significantly different from post-training measures, suggesting that training benefits had been largely retained, consistent with previous studies using a similar test and training protocol [11,12,27].

**Benefits of training the CI ear only for phoneme recognition**

**CI-only listening**

Consistent with previous computer-based training studies for English-speaking [5,6,7,8,9,10,26], German-speaking [14] and Mandarin-speaking CI patients [27], the present study found significant benefits for phonemic contrast training in French CI patients. With CI-only listening, the mean post-training benefit across all participants was 19.1 and 15.9 percentage points for vowel and consonant recognition, respectively. Post-training improvements with the present French participants were comparable to those reported in previous computer-based CI training studies using similar test and training protocols. Fu et al. [26] reported mean training benefits of 15.8 and 13.5 percentage points for vowel and consonant recognition in adult English-speaking CI participants, respectively. Wu et al. [27] reported mean training benefits of 21.7 and 19.5 percentage points for vowel and consonant recognition in pediatric Mandarin-speaking CI (n=7) and hearing aid (n=3) listeners. Thus, the phonemic contrast training used in the present and previous studies [5,6,7,26,27] appears to produce consistent training benefits in terms of vowel and consonant recognition, even for experienced CI patients.

**Binaural listening**

For bilateral and bimodal CI patients, training with a single CI ear generalized to significantly improved binaural phoneme recognition. For bilateral CI patients, the mean post-training improvement was 17.6 and 11.9 percentage points for binaural vowel and consonant recognition, respectively. For bimodal CI patients, the mean post-training improvement was 22.1 and 14.1 percentage points for binaural vowel and consonant recognition, respectively. There was no significant difference in post-training improvements between CI-only and binaural listening, consistent with Zhang et al. [12].

Yoon et al. [17,20] found that the greatest binaural benefit was observed when the performance asymmetry across ears was reduced. For the present bilateral CI patients, mean CI-only vowel recognition was only 2.4 percentage points better with the better ear than with the poorer ear, with across-ear asymmetry as high as 8.3 points. The mean post-training improvement for vowel recognition in bilateral CI patients was quite similar between CI-only (20.4 points) and binaural listening (17.6 points). For bimodal CI patients, performance with the non-implanted ear alone was not measured, but would be expected to be poorer than with the CI ear given the limited frequency extent. As such, substantial performance asymmetry across ears would be expected in bimodal patients. Training with the CI ear alone yielded similar post-training improvements for CI-only (19.8 and 17.1 points for vowel and consonant recognition, respectively) and binaural listening (23.3 and 17.3 points for vowel and consonant recognition, respectively).

Taken together, training the poorer ear in bilateral CI patients or the CI ear alone in bimodal CI patients appeared to only increase the performance asymmetry across ears. For bilateral CI patients, the mean pre-training binaural benefit (binaural performance relative to the better CI ear) for vowel recognition was 9.3 points; the mean post-training binaural benefit was 12.8 points. This suggests that training with the poorer ear alone increased both the performance asymmetry across ears and the binaural benefit. For bimodal patients, the mean pre-training binaural benefit (binaural performance relative to the CI ear) was 9.7 and 8.5 points for vowel and consonant recognition, respectively; the mean post-training binaural benefit was 9.2 and 2.3 points for vowel and consonant recognition, respectively. Here, training the CI ear alone increased the performance asymmetry across ears, but reduced binaural benefit, especially for consonant recognition. In both cases, while training the CI ear alone resulted in better phoneme recognition for both CI-only and binaural listening, it is unclear whether binaural training would have elicited similar or better improvements in CI-only listening, binaural listening, and binaural benefit. For example, Chavant et al. [28] found that for simulations of bimodal listening, including low-frequency acoustic information (even though unintelligible) during training benefited perception of spectro-temporally degraded speech.

**Benefits of training the CI ear only for SRTs in noise**

**CI-only listening**

The mean improvement in CI-only SRTs across unilateral and bimodal CI listeners was 6.5 dB, which was surprising. It is possible that procedural learning may have played a role in the training benefit. For the unilateral group, SRTs were significantly lower for test run 3 than run 1 (Table 8). Also, there were only 100 French HINT sentences in the stimulus set. During each test trial, a sentence was randomly selected from the stimulus set, without replacement. However, given the number of conditions (3 test times x 2 listening modes x 3 repeats), it is likely that some sentences were repeated across test runs and/or test sessions. Given that listeners only had to correctly repeat 50% the words to reduce the SNR (Rule 3 from Chan et al., 2008 [25]), previous exposure to sentences may have been beneficial. However, the mean pre-training CI-only SRT across unilateral and bimodal CI listeners was 10.9 dB. This is comparable to the mean SRT (10 dB) in unilateral CI patients using 260 English HINT sentences [29] reported by Fu and Nogaki [30], but higher than the mean SRT (6.5 dB) reported by Oba et al. [11]. This suggests that repeated exposure may not have been beneficial for baseline SRTs in the present unilateral and bimodal participants. Pre-training CI-only SRTs could not be obtained in 2 unilateral and 2 bimodal CI patients, but were moderately good for the post-training and follow-up tests (Fig 7). This suggests that some perceptual learning may have occurred for these patients after the phonemic contrast training. However, it is difficult to rule out procedural learning.

The adaptive SRT procedure may have been problematic for some patients. Under the test protocol, the initial SNR was 10 dB. If the listener did not repeat 50% or more of the words from the first sentence correctly, the SNR was increased by the initial step size of 4 dB; this continued until reaching the maximum SNR of 25 dB (i.e., four opportunities to correctly identify 50% or more of the words in sentences before the test run was aborted). It is possible that this approach contributed to the failure in obtaining pre-training SRTs for 2 of the unilateral and 2 of the bimodal CI patients and it may have contributed to instances of aberrantly high SRTs for some test runs in other patients. Note that test lists were randomly selected across patients, test runs, and test points, so it seems unlikely that overly high SRTs were associated with a particular test list. In previous studies by Fu and colleagues using English HINT sentences [11], such difficulties in obtaining SRTs in unilateral CI patients using the same method were not observed.

It is also possible that the present French recordings of the French-Canadian HINT sentences from Vaillancourt et al. [24] gave rise to a different intelligibility for some sentences. Note that the mean SRT across both talkers (-5.3 dB) in the present native French NH listeners was lower (better) than reported by Vaillancourt et al. [24] in French-Canadian NH listeners (-3.0 dB). Note that Rule 3 was used in the present study, where listeners had to identify ≥50% of words in sentences to reduce the SNR [25], as opposed to Rule 1 in Vaillancourt et al. [24], where listeners had to identify 100% of words in sentences to reduce the SNR. Also, SRTs were measured in sound field in the present study versus headphones in Vaillancourt et al. [24].

**Binaural listening**

While pre-training CI-only SRTs <25 dB could not be measured in 2 bilateral CI and 2 bimodal CI participants, pre-training binaural SRTs could be measured in all bilateral CI participants and all but one of the bimodal CI participants. This suggests a strong bimodal benefit even for pre-training SRTs, or maybe more accurately, a strong monaural deficit. As CI-only bimodal and bilateral performance was acutely tested and was not the everyday listening mode for these participants, monaural SRTs may have been particularly difficult. Combined with the factors described above (e.g., the test method where the first sentence was repeatedly tested until the listener could identify 50% or more words or until the SNR >25 dB, whichever came first), monaural SRTs may have especially elevated. Note that binaural performance was measured first in bilateral and bimodal participants to allow for procedural learning within the everyday binaural listening mode. It is possible that this task familiarization was not helpful with the subsequent pre-training measures with monaural listening.

For the bimodal CI patients in whom pre-training SRTs <25 dB could be obtained, mean post-training SRTs improved by 7.0 and 6.9 dB for CI-only and binaural listening, respectively. For these same participants, the mean post-training binaural benefit (relative to the CI ear) was quite small, suggesting that the training primarily improved monaural performance in the CI ear, rather than binaural performance. Due to time constraints, CI-only SRTs were not obtained in bilateral CI patients, and without the monaural SRTs, the binaural benefit (relative to the better CI ear) cannot be estimated. However, binaural SRTs <25 dB were obtained in all bilateral CI patients, where mean post-training SRTs improved by 5.7 dB.

**Other observations**

Compliance with the training protocol was good, with an average of 10.3 training hours completed during the one-month training period. Although participants were instructed to immediately contact the experimenters if they had any problems installing or using the software, no problems were observed. Most participants found that the software was easy to use, and that the training was easy to perform, although some mentioned it got a bit boring after a while. The home training was particularly opportune given the COVID-19 pandemic. As such, the French Angelsound software is a very useful rehabilitation tool when in-person appointments are difficult or impossible.

There was no significant training benefit for any of the SSQ scores (Fig 9). This suggests that the training benefits observed with the lab-based outcome measures did not generalize to the listening scenarios captured by the SSQ questionnaire. Note that the questionnaire reflected hearing abilities over a longer term for a variety of scenarios with patients’ everyday listening condition (one CI for unilateral patients, two CIs for bilateral patients, and a CI and contralateral hearing aid for bimodal patients). The speech outcome measures in quiet or with steady noise do not reflect the complex listening environments encountered in everyday life which the SSQ questionnaire seeks to address.

While significant benefits were observed for the present computer-based phonemic contrast training, other approaches have also been shown to be beneficial. Previous studies have shown significant training benefits using a connected discourse training protocol [31,32]; similar benefits were observed between labor-intensive in-person and computer-based connected discourse training [33]. Oba et al. [11] found significant benefits for digits-in-noise training. While the present study showed significant benefits for bilateral and bimodal CI patients for both CI-only and binaural listening when training with a single CI ear, it is possible that binaural training may show similar benefits or more. Note that all stimuli were presented directly in front of the listener when testing or training. Binaural training with spatial cues may help segregate competing speech.

While the present training outcomes are promising, there were only 15 participants in the present study. The limited number of participants makes it difficult to draw strong conclusions, especially for binaural benefits with training. As such, we regard this as a worthwhile pilot study. The stimuli and method for measuring SRTs may have been problematic for some listeners. Using different outcome measures (e.g., the French Matrix test from Jansen et al. [34] or the FrBio from Bergeron et al. [35] may help to stabilize pre-training SRTs and better observe training benefits. Still, the present data suggest that the phonemic contrast training in French AngelSound may benefit French-speaking CI patients. Computer-based home training may be a useful alternative and/or complement to in-person rehabilitation, which is time-intensive and may not always be possible under certain circumstances (e.g., the COVID-19 pandemic and lockdowns). The computer-based training also offers a consistent training experience, which may not always be the case across CI rehabilitation centers, where approaches and speaking voices may differ. A research version of French AngelSound was used in the present study, which restricted access to many other training modules (e.g., familiar word identification, environmental sound identification, speech in noise perception, music perception, etc.). The rich training materials in the full version of the software may further benefit French-speaking CI patients, and we plan to further evaluate the benefits of French AngelSound in a larger group of CI patients.

**Acknowledgments**

We thank all the participants for their time and effort. We also thank Julie Foucaut, Marion Marguet-Isambert, Antoine Papazian and Dr. Charles Aussedat for their assistance with recording the speech stimuli for French AngelSound, and Mathieu Robier and Flavie Lemieux for their help with the study.

**Author Contributions**

Conceptualization: John J. Galvin III, Qian-jie Fu, David Bakhos, Sandrine Kerneis,

Data curation: Sandrine Kerneis

Formal analysis: John J. Galvin III

Investigation: Sandrine Kerneis, Jean Baqué

Methodology: John J. Galvin III, Qian-jie Fu, Stephanie Borel, David Bakhos, Sandrine Kerneis,

Software: Qian-jie Fu

Project administration: Sandrine Kerneis, John J. Galvin III, Qian-jie Fu, David Bakhos

Supervision: Sandrine Kerneis, John J. Galvin III, Qian-jie Fu, David Bakhos

Validation: Sandrine Kerneis, John J. Galvin III, Stephanie Borel, Qian-jie Fu,

Visualization: John J. Galvin III

Writing – original draft: Sandrine Kerneis, John J. Galvin III

Writing – revision: John J. Galvin III, Sandrine Kerneis,

Writing – review & editing: Sandrine Kerneis, John J. Galvin III, Jean Baqué, Qian-jie Fu, Stephanie Borel, David Bakhos

**References**

[1] Spivak LG, Waltzman SB. Performance of cochlear implant patients as a function of time. *J Speech Hear Res*. 1990;33(3):511-519. doi:10.1044/jshr.3303.511
[2] Loeb GE, Kessler DK. Speech recognition performance over time with the Clarion cochlear prosthesis. Ann Otol Rhinol Laryngol Suppl. 1995 Sep;166:290-2.
[3] Dorman MF, Loizou PC. Changes in speech intelligibility as a function of time and signal processing strategy for an Ineraid patient fitted with continuous interleaved sampling (CIS) processors. *Ear Hear*. 1997;18(2):147-155. doi:10.1097/00003446-199704000-00007
[4] Pelizzone M, Cosendai G, Tinembart J. Within-patient longitudinal speech reception measures with continuous interleaved sampling processors for ineraid implanted subjects. *Ear Hear*. 1999;20(3):228-237. doi:10.1097/00003446-199906000-00005
[5] Fu QJ, Galvin J, Wang X, Nogaki G. Effects of auditory training on adult cochlear implant patients: A preliminary report. Cochlear Implants Int. 2004 Sep;5 Suppl 1:84-90. doi: 10.1179/cim.2004.5.Supplement-1.84.
[6] Fu QJ, Galvin JJ 3rd. Computer-assisted speech training for cochlear implant patients: feasibility, outcomes, and future directions. Semin Hear. 2007a May 1;28(2):10.1055/s-2007-973440. doi: 10.1055/s-2007-973440.
[7] Fu QJ, Galvin JJ 3rd. Perceptual learning and auditory training in cochlear implant recipients. Trends Amplif. 2007b Sep;11(3):193-205. doi: 10.1177/1084713807301379.
[8] Fu QJ, Galvin JJ 3rd. Maximizing cochlear implant patients' performance with advanced speech training procedures. Hear Res. 2008 Aug;242(1-2):198-208. doi: 10.1016/j.heares.2007.11.010.
[9] Stacey PC, Summerfield AQ. Comparison of word-, sentence-, and phoneme-based training strategies in improving the perception of spectrally distorted speech. J Speech Lang Hear Res. 2008 Apr;51(2):526-38. doi: 10.1044/1092-4388(2008/038).
[10] Stacey PC, Raine CH, O'Donoghue GM, Tapper L, Twomey T, Summerfield AQ. Effectiveness of computer-based auditory training for adult users of cochlear implants. Int J Audiol. 2010 May;49(5):347-56. doi: 10.3109/14992020903397838.
[11] Oba SI, Fu QJ, Galvin JJ 3rd. Digit training in noise can improve cochlear implant users' speech understanding in noise. Ear Hear. 2011 Sep-Oct;32(5):573-81. doi: 10.1097/AUD.0b013e31820fc821.
[12] Zhang T, Dorman MF, Fu QJ, Spahr AJ. Auditory training in patients with unilateral cochlear implant and contralateral acoustic stimulation. Ear Hear. 2012 Nov-Dec;33(6):e70-9. doi: 10.1097/AUD.0b013e318259e5dd
[13] Henshaw H, Ferguson MA. Efficacy of individual computer-based auditory training for people with hearing loss: a systematic review of the evidence. PLoS One. 2013 May 10;8(5):e62836. doi: 10.1371/journal.pone.0062836.
[14] Schumann A, Serman M, Gefeller O, Hoppe U. Computer-based auditory phoneme discrimination training improves speech recognition in noise in experienced adult cochlear implant listeners. Int J Audiol. 2015 Mar;54(3):190-8. doi: 10.3109/14992027.2014.969409.
[15] Cheng X, Liu Y, Shu Y, Tao DD, Wang B, Yuan Y, Galvin JJ 3rd, Fu QJ, Chen B. Music training can improve music and speech perception in pediatric Mandarin-speaking cochlear implant users. Trends Hear. 2018 Jan-Dec;22:2331216518759214. doi: 10.1177/2331216518759214.
[16] Fu QJ, Shannon RV, Galvin JJ 3rd. Perceptual learning following changes in the frequency-to-electrode assignment with the Nucleus-22 cochlear implant. J Acoust Soc Am. 2002 Oct;112(4):1664-74. doi: 10.1121/1.1502901.
[17] Yoon YS, Li Y, Kang HY, Fu QJ. The relationship between binaural benefit and difference in unilateral speech recognition performance for bilateral cochlear implant users. Int J Audiol. 2011a Aug;50(8):554-65.
[18] Yoon YS, Liu A, Fu QJ. Binaural benefit for speech recognition with spectral mismatch across ears in simulated electric hearing. J Acoust Soc Am. 2011b Aug;130(2):EL94-100.
[19] Svirsky MA, Fitzgerald MB, Sagi E, Glassman EK. Bilateral cochlear implants with large asymmetries in electrode insertion depth: implications for the study of auditory plasticity. Acta Otolaryngol. 2015 Apr;135(4):354-63.
[20] Yoon YS, Shin YR, Gho JS, Fu QJ. Bimodal benefit depends on the performance difference between a cochlear implant and a hearing aid. Cochlear Implants Int. 2015 May;16(3):159-67. doi: 10.1179/1754762814Y.0000000101.
[21] Fu QJ, Galvin JJ 3rd, Wang X. Integration of acoustic and electric hearing is better in the same ear than across ears. Sci Rep. 2017 Oct 2;7(1):12500.
[22] Noble W, Jensen NS, Naylor G, Bhullar N, Akeroyd MA. A short form of the Speech, Spatial and Qualities of Hearing scale suitable for clinical use: The SSQ12. Int J Audiol. 2013;52(6):409-412. doi:10.3109/14992027.2013.781278
[23] Plomp R, Mimpen AM. Speech-reception threshold for sentences as a function of age and noise level. J Acoust Soc Am. 1979 Nov;66(5):1333-42. doi: 10.1121/1.383554
[24] Vaillancourt V, Laroche C, Mayer C, Basque C, Nali M, Eriks-Brophy A, Soli SD, Giguère C. Adaptation of the HINT (hearing in noise test) for adult Canadian Francophone populations. Int J Audiol. 2005 Jun;44(6):358-69. doi: 10.1080/14992020500060875.
[25] Chan JC, Freed DJ, Vermiglio AJ, Soli SD. Evaluation of binaural functions in bilateral cochlear implant users. Int J Audiol. 2008 Jun;47(6):296-310. doi: 10.1080/14992020802075407.
[26] Fu QJ, Galvin JJ III, Wang X, Nogaki G. Moderate auditory training can improve speech performance of adult cochlear implant users. Acoust Res Lett Online. 2005;6:106-111.
[27] Wu JL, Yang HM, Lin YH, Fu QJ. Effects of computer-assisted speech training on Mandarin-speaking hearing-impaired children. Audiol Neurootol. 2007;12(5):307-12. doi: 10.1159/000103211.
[28] Chavant M, Hervais-Adelman A, Macherey O. Perceptual learning of vocoded speech with and without contralateral hearing: implications for cochlear implant rehabilitation. J Speech Lang Hear Res. 2021 Jan 14;64(1):196-205. doi: 10.1044/2020_JSLHR-20-00385.
[29] Nilsson M, Soli SD, Sullivan JA. Development of the Hearing in Noise Test for the measurement of speech reception thresholds in quiet and in noise. J Acoust Soc Am. 1994 Feb;95(2):1085-99. doi: 10.1121/1.408469.
[30] Fu QJ, Nogaki G. Noise susceptibility of cochlear implant users: the role of spectral resolution and smearing. J Assoc Res Otolaryngol. 2005;6(1):19-27. doi:10.1007/s10162-004-5024-3
[31] Faulkner A, Rosen S, Green T. Comparing live to recorded speech in training the perception of spectrally shifted noise-vocoded speech. J Acoust Soc Am. 2012 Oct;132(4):EL336-42. doi: 10.1121/1.4754432.
[32] Green T, Rosen S, Faulkner A, Paterson R. Adaptation to spectrally-rotated speech. J Acoust Soc Am. 2013 Aug;134(2):1369-77. doi: 10.1121/1.4812759.
[33] Green T, Faulkner A, Rosen S. Computer-based connected-text training of speech-in-noise perception for cochlear implant users. Trends Hear. 2019 Jan-Dec;23:2331216519843878. doi: 10.1177/2331216519843878.
[34] Jansen S, Luts H, Wagener KC, Kollmeier B, Del Rio M, Dauman R, James C, Fraysse B, Vormès E, Frachet B, Wouters J, van Wieringen A. Comparison of three types of French speech-in-noise tests: A multi-center study. Int J Audiol. 2012 Mar;51(3):164-73. doi: 10.3109/14992027.2011.633568.
[35] Bergeron F, Berland A, Fitzpatrick EM, Vincent C, Giasson A, Leung Kam K, Chafiq W, Fanouillère T, Demers D. Development and validation of the FrBio, an international French adaptation of the AzBio sentence lists. Int J Audiol. 2019 Aug;58(8):510-515. doi: 10.1080/14992027.2019.1581950.
