# Supplementary material for: Preliminary evaluation of computer-assisted home training for French cochlear implant recipients
Source: PLoS One. 2023 Apr 28;18(4):e0285154. doi: 10.1371/journal.pone.0285154 (PMC10146517; doi:10.1371/journal.pone.0285154)
Supplement: Attachment — Submitted filename: Response to reviewers.docx [file pone.0285154.s006.docx]

To the editor,

We thank the reviewers and the editor for their helpful comments and suggestions and have revised the MS with these comments in mind. Changes in the marked-up copy are shown using track changes. Major changes include:

- Revised analysis of the vowel and consonant data to include all test runs, as suggested by Rev. 1. The attendant figures and text have been modified accordingly.
- Revised figure and analysis of the binaural advantage data.
- Revised SSQ analysis as suggested by Rev. 1

We feel that the changes make the paper more straightforward and we hope you find these changes acceptable. Please contact me if you need further information. Below, we respond to specific reviewer comments.

Sincerely,

Sandrine Kerneis

Reviewer #1

While adequately addressing some key issues the revisions to the ms have introduced new problems. A reasonable case can be made that stable performance over repeated testing pre-training indicates that comparisons between pre- and post-training are unlikely to be much affected by procedural learning. However, the approach of including test run as a factor and then excluding some post-training runs on a post-hoc basis does not seem appropriate to me. Excluding the post-training runs with poorer performance would inevitably seem to result in bias towards finding beneficial effects of training.

>> We have redone all vowel and consonant data analysis and figures with all data included. As the story doesn’t change, this is a much cleaner presentation. We thank you for the suggestion.

The removal of the analysis of the sentence data is not reflected in some parts of the ms. The Abstract mentions sentence in noise recognition as an outcome measure and reports only that performance improved after training, implying that this included sentence recognition. The first sentence of the Discussion states that there were significant benefits for sentence recognition in noise.

>> We now specify in the Abstract: “For all participants, post-training CI-only vowel and consonant recognition scores significantly improved after phoneme training with the CI ear alone. For bilateral and bimodal CI users, binaural vowel and consonant recognition scores also significantly improved after training with a single CI ear.”

And later in the Discussion: “The computer-based home training showed significant benefits for vowel and consonant recognition for the present French CI participants, consistent with previous home-training studies with English-, German- and Mandarin-speaking CI users [5,6,7,8,9,10,11,12,14,15].”

Other points

L206 – ‘Data were averaged across all runs’ - This is inconsistent with test run being included as a factor in the analyses.

>> As noted above, we now include all data in the analyses and figures, so this statement is now correct.

L238 – The reference to NH testing seems out of place here. I recommended omission of the NH data, but if they are going to be included their purpose needs to be made clear. Also, in the Results it is stated that SRTs were obtained from 13 listeners.

>> We prefer to retain the NH data, as it provides a point of comparison for the CI data in Figs 3-5. We have deleted the sentence, but have added to the Participants section: “Sixteen adults with normal hearing (NH; mean age at testing = 42.5±17.9 years) were also recruited as experimental controls for the CI test data; vowel and consonant recognition were measured in 16 listeners with NH, and sentence recognition in noise was measured in 13 listeners with NH.”

And later in the Results” “However, CI participants’ SRTs remained much poorer than mean SRTs for listeners with NH (-5.3 ± 2.2 dB), even after training.”

L253 – ‘high-quality recordings…’ – this is stated earlier.

>> The sentence has been deleted.

L353 – Make clear whether they were asked or if these were spontaneous requests.

>> Revised as: “Nonetheless, 66% of participants spontaneously asked if they could keep the training software after the experiment was finished; these requests were honored.”

L374 – This suggests that binaural performance was assessed in the unilateral group.

>> Revised as: “LMM analysis was also performed on the binaural vowel score data, with group (bilateral, bimodal), test point (pre, post, follow-up), and test run (1, 2, 3, 4) as fixed factors and participant as a random factor.”

L380 – This should presumably say ‘vowel’ rather than ‘consonant’

>> Thanks. This section has been greatly modified, and we corrected the mistake.

L399 and similarly at L441 - It’s not clear exactly which analysis is being referred to here. Were additional analyses performed on differences between pre- and post-training performance?

>> For vowels, revised as: “LMM analyses were performed on the CI-only vowel training benefit data, with group (unilateral, bilateral, bimodal), test point (post, follow-up), and test run (1, 2, 3) as fixed factors and participant as a random factor. Results showed no significant main effects. LMM analyses were also performed on the binaural vowel training benefit data, with group (bilateral, bimodal), test point (post, follow-up), and test run (1, 2, 3) as fixed factors and participant as a random factor. Results showed no significant main effects.”

For consonants, revised as: “LMM analysis were performed on the CI-only consonant training benefit data, with group (unilateral, bilateral, bimodal), test point (post, follow-up), and test run (1, 2, 3) as fixed factors and participant as a random factor; results showed no significant main effects. LMM analysis were also performed on the binaural consonant training benefit data, with group (bilateral, bimodal), test point (post, follow-up), and test run (1, 2, 3) as fixed factors and participant as a random factor; results showed no significant main effects.”

L428 – ‘all 3 test runs’ – earlier it says there were 2-3 runs

>> This section has been greatly modified, and we corrected the mistake.

L452 – It’s not clear here exactly which data is included in this analysis but in any case I don’t think that comparing benefit between vowel and consonant tasks is very meaningful or useful. No rationale is provided to expect a difference and given that the tasks differ in various ways such as the numbers of response options and the starting levels of performance I don’t think that finding a significant difference in percentage point benefit would really be informative about changes in underlying perceptual processes.

>> We have deleted this section.

L473 – SNR rather than SRT

>> This has been corrected.

L478 – Again, I’m not convinced that this analysis is really worthwhile.

>> We prefer to retain this section, as we state at the end of the Introduction: “We were interested in whether training a single CI would also improve binaural performance in bilateral and bimodal CI users and, if so, whether binaural improvements would be super-additive (greater than CI-only improvements) or simply driven by CI-only improvements.” We have modified Fig 6 to explicitly show the binaural advantage at each test point, and we have modified the LMM to compare binaural advantage across groups, tests, and test points.

L513 – Although it’s unlikely to change any of the conclusions, given that it doesn’t appear to be of interest whether scores are higher overall for any particular subsection, it would appear more natural to analyse each subsection separately, rather than to treat subsection as a factor.

>> We have performed the suggested analysis and have modified this section accordingly.

L599 – ‘somewhat larger improvements’ – this seems to be comparing the magnitude of an increase in percent correct in a closed-set phoneme recognition task with a change in SRT which doesn’t really seem to make sense.

>> We have deleted this section of the paragraph.

Reviewer #2

I reviewed this paper originally as reviewer 2.

The work aims to evaluate French Angelsound in 15 people with CI (5 unilateral, 5 bilateral, 5 bimodal). They performed 10 hours training over one month.

Thank you to the authors for considering and mostly including my suggestions in your revised manuscript. This is now a v well written manuscript with a clear abstract; it will contribute to the literature. I love your ‘Other observations’ paragraphs.

There are only 3 things I suggest and they are word changes/copy editing, so I will not need to rereview:

Line 142. I think it’s a bit odd to put ‘some of our research questions were’. Maybe just put that those were your primary research questions.

Line 143 repetition of word ‘would’

>> In response to both comments. Revised as: “We were interested in whether training a single CI would also improve binaural performance in bilateral and bimodal CI users and, if so, whether binaural improvements would be super-additive (greater than CI-only improvements) or simply driven by CI-only improvements”

line 345 try and ensure there is not a line break as it currently looks like the SRTs were 5.3 on quick glance – can you remove a space perhaps?

>> This has been corrected.
